# Using Expression Profiles of *Caenorhabditis elegans* Neurons To Identify Genes That Mediate Synaptic Connectivity

**DOI:** 10.1371/journal.pcbi.1000120

**Published:** 2008-07-11

**Authors:** Leehod Baruch, Shalev Itzkovitz, Michal Golan-Mashiach, Ehud Shapiro, Eran Segal

**Affiliations:** 1 Department of Computer Science and Applied Mathematics, Weizmann Institute of Science, Rehovot, Israel; 2 Department of Biological Chemistry, Weizmann Institute of Science, Rehovot, Israel; Indiana University, United States of America

## Abstract

Synaptic wiring of neurons in *Caenorhabditis elegans* is largely invariable between animals. It has been suggested that this feature stems from genetically encoded molecular markers that guide the neurons in the final stage of synaptic formation. Identifying these markers and unraveling the logic by which they direct synapse formation is a key challenge. Here, we address this task by constructing a probabilistic model that attempts to explain the neuronal connectivity diagram of *C. elegans* as a function of the expression patterns of its neurons. By only considering neuron pairs that are known to be connected by chemical or electrical synapses, we focus on the final stage of synapse formation, in which neurons identify their designated partners. Our results show that for many neurons the neuronal expression map of *C. elegans* can be used to accurately predict the subset of adjacent neurons that will be chosen as its postsynaptic partners. Notably, these predictions can be achieved using the expression patterns of only a small number of specific genes that interact in a combinatorial fashion.

## Authors Summary

Synaptic wiring in the nematode *Caenorhabditis elegans* is largely invariant between individuals, suggesting that this wiring is genetically encoded. This is in essence the chemoaffinity hypothesis suggested by Roger Sperry. However, proving this hypothesis in model organisms and detecting the identities of the genes that determine the presence or absence of synaptic connections is a major challenge. *C. elegans* provides a unique opportunity to examine this hypothesis due to the availability of both its neuronal wiring diagram and neuronal gene expression map. In this study we show that the neuronal gene expression profiles can be used to predict the subset of adjacent neurons that each neuron will connect to with good accuracy. We further identify a small set of putative genes on both sides of the synapses that interact in a combinatorial fashion and mediate the neuronal partner selection process. The modular design in which a small set of components is reutilized throughout the network is common with other known biological systems and raises the possibility of a similar design in neuronal networks of more complex organisms.

## Introduction

The nervous system of *Caenorhabditis elegans* has exactly 302 neurons with a simple gross morphology, often having only a single, unbranched process. Processes run together in parallel bundles, forming synapses to adjacent processes. The neuronal bodies and their processes are found in characteristic positions and similar sets of synaptic connections are seen in different individuals and among sets of homologous cells (e.g., cells that are bilaterally symmetrical to each other in the worm's body) [1]. Furthermore, most of the neurons are connected to a subset of about 50% of the neurons that are in physical proximity to them and this subset is fairly constant from animal to animal [2],[3]. These observations raise the fundamental question in neuroscience: What are the rules that govern nervous system connectivity and how are these rules encoded in the genome?

The development of the nervous system can be divided into three phases: The generation of the correct cells in the right temporal and spatial locations, the outgrowth of nerve processes, and the formation of synapses. The first phase is determined by the lineage of the organism, which positions the neurons at the right temporal and spatial locations. The second phase depends mostly on the growth cone which migrates through the animal, spinning out the nerve process behind it. The third phase depends on short range communication and is feasible only between neurons that are in physical proximity. All of these phases show a high degree of specificity [1],[2].

Here, we focus on the third phase in which a neuron “chooses” its synaptic partners from among the neurons that are in physical proximity to it. A classical hypothesis for this phase with many empirical proofs is Sperry's chemoaffinity hypothesis [4]–[6], which states that the wiring is “activity-independent,” i.e., that each neuron links to a postsynaptic target by selective attachment mediated by specific chemical molecular identifiers. These molecular identifiers are encoded in the genome [7], label the neurons, and determine their chemical affinity. Candidate genes which may constitute the molecular identifiers are the *Dscam* gene in *drosophila* [8] and the Protocadherin (*Pcdh*) proteins in humans [9]. In *C. elegans*, the most unequivocal proof for the existence of such molecular identifiers was demonstrated for a single neuron (HSNL) [10], where it was shown that the transmembrane proteins *syg-1* and *syg-2*, members of the immunoglobulin superfamily, bind together and guide the neuron to form the correct synapses.

The relationship between connectivity and gene expression in *C. elegans* was recently explored in two studies. Kaufman et al. [11] was the first study to demonstrate a correlation between gene expression and neuronal connectivity using a covariation correlation analysis. They also showed that the expression signature of each neuron can be used to predict its outgoing connectivity signature using the *k*-nearest neighbors method, i.e., neurons that express similar sets of genes tend to choose similar sets of synaptic partners. A similar result was separately shown for the incoming connectivity. They used feature selection to find a small set of genes whose expression carries most of the neuronal connectivity information. However, their approach does not provide predictions on the way in which these genes interact to mediate synaptic connectivity.

In a closely related study, Varadan et al. [12] applied an entropy minimization approach to identify sets of synergistically interacting genes whose joint expression pattern predicts the existence of a synapse with minimum uncertainty. They provide a single rule, composed of two genes in the presynaptic region and two genes in the postsynaptic region whose joint expression predicts the existence of a synapse with minimum uncertainty. This rule achieved significantly smaller entropy than that expected by chance, but its predictive ability was not examined in a cross-validation scheme.

A common feature in both of the above studies [11],[12] is the attempt to predict the formation of a chemical synapse between any pair of neurons in the worm based on the expression pattern of the genes, regardless of their spatial location. Here, we propose to integrate the spatial locations of neurons into this prediction task, by limiting the predictions to pairs of neurons that are certain to be in physical proximity to each other in the worm's body (since they are connected by chemical or electrical synapses). By doing so, we shift the focus from genes whose expression affects synaptic connectivity through mechanisms such as lineage, axonal guidance and neuronal migration to genes whose expression has a role in the crosstalk of the neurons in the final stage of the chemical synapse formation when neurons identify their designated partners.

Our study has two complementary goals. First, we wish to explore whether the gene expression signature of the neurons carries significant information on the subset of adjacent neurons that are chosen as their postsynaptic partners. Second, we wish to find a subset of genes and specific rules of interactions among them that with high confidence predict the choice of chemical synaptic partners. We combine the gene expression patterns of neurons with the neuronal wiring diagram, and apply a probabilistic learning algorithm for detecting the subset of relevant genes and their combinatorial logic, while incorporating the physical proximity of the neurons.

Our results confirm that neuronal gene expression can be used to accurately predict the choice of synaptic partners and that only a few genes with specific interaction patterns are sufficient to make these predictions. We suggest that this small number of genes imply that there may be a general genetic mechanism that wires the nervous system of the worm and that deeper understanding of this mechanism may contribute to the understanding of the development of nervous systems in higher organisms.

## Results

**Figure 1:**
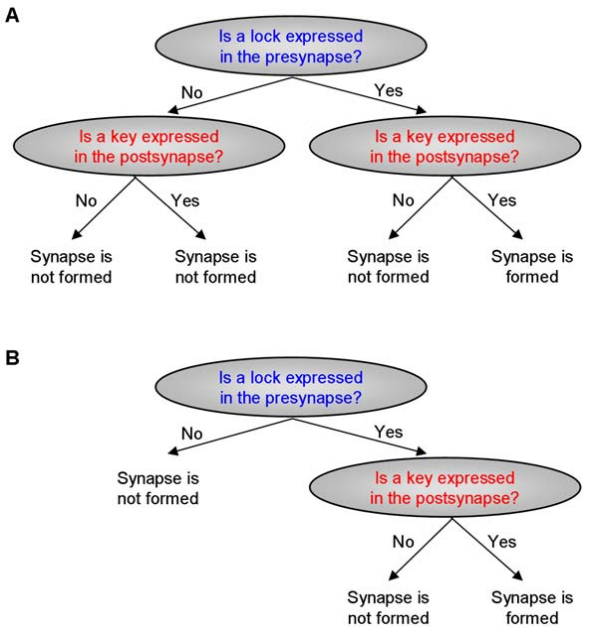
Context Specific Independencies Reduce the Complexity of the Model and Make It Easier To Interpret. (A) A complete decision tree for the simplified example of a lock-and-key molecular identifiers mechanism: only when the presynaptic neuron expresses a lock molecule and the postsynaptic neuron expresses a key molecule, a synapse is formed between them. (B) A simpler decision tree that captures the same logic but exhibits context specific independence. In the context in which the lock is not expressed in the presynaptic neuron, the formation of a synapse between adjacent neurons is independent of the expression of the key in the postsynaptic neuron.

Our goal is to model the dependence of the chemical synapse formation on the expression patterns of the genes in the neurons. To this end, we introduce a variable representing the chemical synapse formation between neurons and try to predict its value based on a stochastic logical function of the expression of the genes in both the presynaptic and postsynaptic neurons. We chose a model that is based on a probabilistic decision tree, which uses the expression pattern of genes in adjacent neurons to regress upon the chemical synapse formation variable. This model has two important virtues which make it suitable for our task. First, it permits context specific independencies: rather than maintaining a complete tree with all the possible splits for gene expression levels, it maintains only the branches which are relevant. For example, consider a simple mechanism of lock-and-key molecular identifiers such that only when the presynaptic neuron expresses a lock molecule and the postsynaptic neuron expresses a key molecule, a synapse would be formed between them (Figure 1A). However, if a neuron does not express the lock then it will not form a synapse onto its neighbors, regardless of the expression of the key. Thus, the decision tree branch that corresponds to the scenario in which the lock is not expressed in the presynaptic neuron should not be split again by the key expression in the postsynaptic neuron (Figure 1B). In this case, in the context in which the lock is not expressed in the presynaptic neuron, the formation of a synapse between adjacent neurons is independent of the expression of the key in the postsynaptic neuron. This way, the context specific independencies reduce the number of model parameters to only those that are relevant, making the model both more intuitive to interpret and easier to robustly learn from the data.

The second virtue of our model is its probabilistic nature, which is important given that both the wiring diagram and the available gene expression patterns are crude and noisy [11]. In addition, although largely constant, the wiring diagram between animals displays some variability, which may be a consequence of a nondeterministic selection of neuronal partners based on their chemical affinities or a consequence of other mechanisms of synaptic plasticity such as Hebb law for activity-dependent synaptic formation [13]. For these reasons, a probabilistic model seems appropriate, since it can account for the noise and inherent variability in the problem.

Our probabilistic decision tree is an instantiation of a probabilistic graphical model, or Bayesian network. Specifically, we chose the tree-structured conditional probability distribution (tree-CPD) that was introduced by Friedman and Goldszmidt [14]. This tree-CPD assigns a conditional probability to every leaf. Thus, every pair of neighboring neurons is mapped to a single leaf based on the genes that they express and the probability of synapse formation between them is obtained from that leaf. For example, in the tree-CPD of Figure 4F, if the postsynaptic neuron expresses hmr-1 and the presynaptic neuron does not expresses npr-1, then the probability of chemical synapse in this direction is 0.92. This probability is independent of akt-1, glr-1, cdh-3, osm-6, and unc-4, although these genes affect the probability of chemical synapse formation in other contexts.

**Figure 2:**
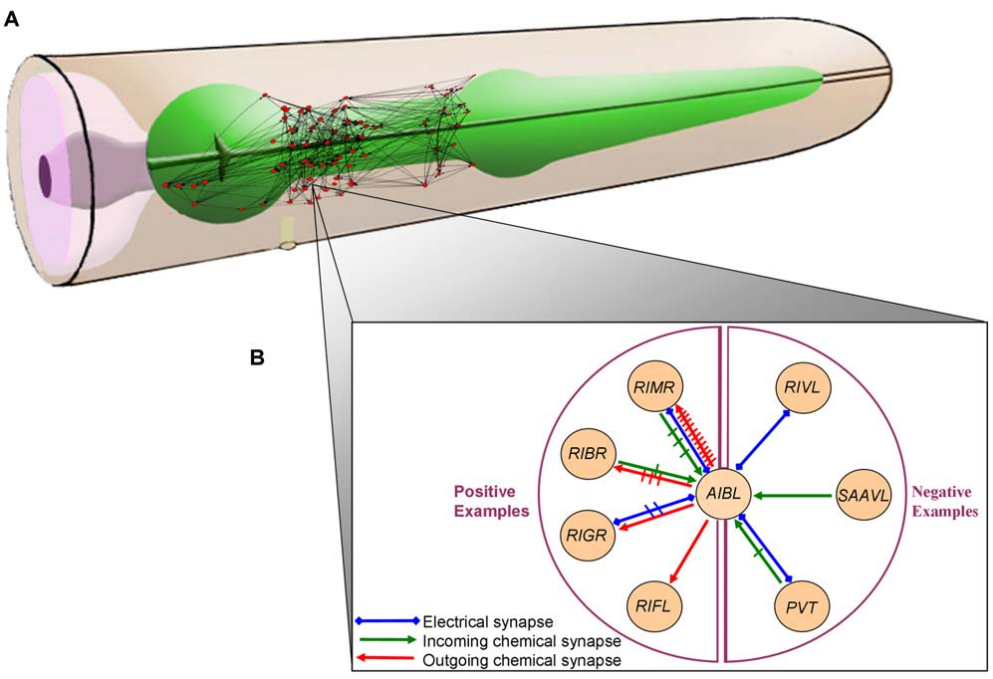
The Neural Network of the *C. elegans* Provides Examples for Learning the Patterns of Synaptic Wiring. (A) A standard schematic of the worm's head (taken from Wormatlas [43]) with a network depiction of a part of *C. elegans*'s neural network on the right side of the nerve ring. Neurons are in their real relative location (data taken from the authors of [44]). (B) An example of a neighborhood of one neuron. The neuron AIBL introduces all types of combinations of synaptic relations with other neurons. For each such combination one neuron has been chosen to demonstrate it. For example the neuron RIVL is the representative of the group of neurons that forms only electrical synapses with AIBL. Each cross on a synapse represents one more additional identical synapse that was observed. The neighborhood of a neuron is defined as the group of neurons that forms a synapse with it (chemical or electrical synapse in either direction). Neurons that are in the same neighborhood must be in spatial proximity in the worm's body. A positive example is created when a neuron “chooses” to be presynaptic to another neuron in its neighborhood and a negative example is created when a neuron “chooses” not to be presynaptic to another neuron in its neighborhood.

We use both the gene expression signature of the neurons and the synaptic connectivity network to learn the model. Since many genes have nearly identical expression patterns, we clustered the neuronal expression patterns of the 251 genes in the dataset into 133 expression classes, thereby removing redundancies in the dataset (see Materials and Methods section). Recall that we wish to focus on the last phase of synaptic connectivity, in which neurons perform crosstalk with each other in order to correctly choose their designated synaptic partners. Thus, ideally, we should choose every ordered pair of neurons that are spatially proximal (such that a chemical synapse could be created between them) at some stage of development as an example to learn from. However, lacking detailed geometric coordinates of the neuronal processes, we use the connectivity pattern itself to approximate the physical proximity of any two neurons. Specifically, we define two neurons as being in the same neighborhood if they are connected by a chemical synapse in either direction or by an electrical synapse (gap junction). According to this definition, neurons in the same neighborhood are certainly close enough to form synapse in either direction (Figure 2). Our approximation may miss negative examples in cases where two neurons that are close enough to form chemical synapse do not form any synapse in either direction. To further validate that our results are not biased due to this approximation, we compared them (below) to the results achieved by applying the same learning process under a more relaxed assumption according to which two neurons are considered spatially proximal if they are both connected by an electrical or chemical synapse to each other or to another neuron in the network.

To learn the tree-CPD model, we used a Bayesian score [15] and a two phase tree-CPD construction heuristic [14]. The Bayesian score exhibits a tradeoff between the fit to the data and the complexity of the model, a desirable property that prevents overfitting. The two phase tree-CPD construction heuristic is designed to prevent the learning process from getting stuck in local minima by scanning the space of tree-CPDs in a way that allows temporary reduction of the score (see Materials and Methods section).

We first tested whether the model learned from this data indeed demonstrates that the gene expression signature of the neurons has predictive power regarding the subgroup of adjacent neurons that will be chosen as the postsynaptic partners of every neuron. We used the tree-CPD as a classifier which predicts the presence or absence of a synapse for each ordered pair of neurons, and extended it by AdaBoost [16], a boosting algorithm designed to improve the accuracy of classifiers. In general, AdaBoost is an iterative algorithm that iteratively learns a new tree-CPD on a reweighed dataset, where the reweighting in each learning iteration is done in a way that shifts the focus from the correctly classified examples (easy examples) to the wrongly classified ones (hard examples). The final classifier is a weighted majority vote of all of the tree-CPDs that were learned (see Materials and Methods section). To assess the quality of the classifier, we compared its accuracy using the standard area under the ROC curve (AUC) for 5-fold cross-validation, to the accuracy obtained for randomized datasets, in which neurons identities were shuffled [11],[12], or in which the examples signs (presence or absence of a synapses) were shuffled (see Materials and Methods section).

**Figure 3:**
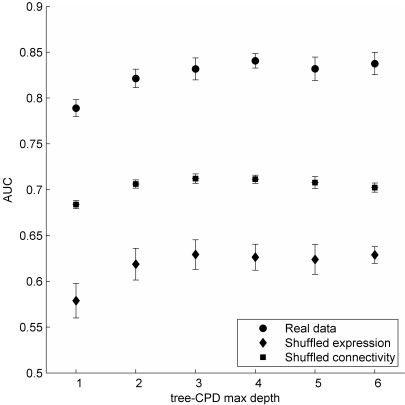
Summary of the Prediction Performance as a Function of the Maximal Depth of the Tree-CPD after 30 AdaBoost Iterations. Standard deviation of the real data was calculated on 50 iterations of 5-fold cross validation, each time for a different division of the data to train and test sets. Standard deviation of the random models was calculated on 50 iterations of 5-fold cross validation, each time for a different shuffling of the data.

We find that our boosted tree-CPD classifier predicts the formation of synapses with an AUC of 0.84±0.008, significantly better than the AUC of 0.71±0.005 achieved on the randomized datasets (Figure 3). The use of boosted decision trees allows us to achieve high performance with shallow tree-CPDs, compared to using nonboosted classifiers (Text S1 and Figure S1). This high performance is independent of the maximal depth of the tree and requires less than 30 boosting iterations to reach maximal performance (Figure S2).

The performance obtained for repeating the same experiment under the relaxed proximity assumption described above was AUC of 0.78±0.01 for the real dataset compared to AUC of 0.64±0.008 for the randomized dataset. Although the performance on both the real and randomized datasets has decreased (due to the 10 fold increase in the number of negative examples while maintaining the same number of positive examples as before), the significance of the results has remained the same. These results therefore show that a probabilistic classifier can predict neuronal connectivity from neuronal expression patterns with good accuracy, thereby achieving the first goal of our study.

**Figure 4:**
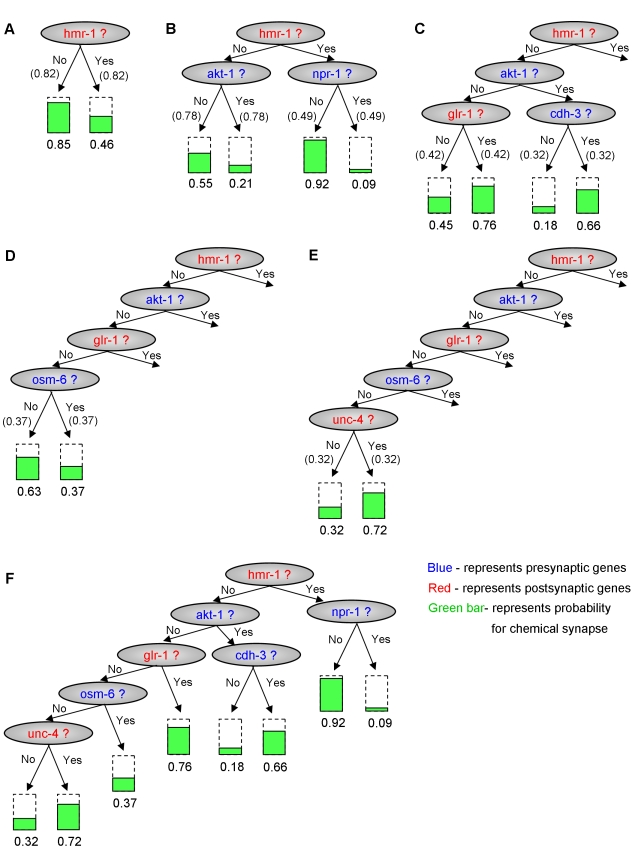
The Highest Confidence Rules That Were Learned in Bootstrap of Tree-CPDs. The highest confidence rules that were learned in bootstrap of tree-CPDs of maximal depth of one (A), two (B), three (C), four (D), and five (E). The confidence of each rule is written in parentheses. (F) The final, most confident, tree-CPD for the chemical synapse. This tree was constructed by combining the rules from (A–E).

We next asked whether we can identify a set of genes and specific rules of interactions among them that explain the choice of chemical synaptic partners with high confidence. The model learned above provides predictions about such putative genes with specific interaction patterns. However, the set of these putative genes and the way they interact may vary for different divisions of the data into train and test sets, raising the question of how confident we are in the set of rules that were learned. To examine the confidence of the rules that were learned, we used a standard nonparametric bootstrap [17] approach of tree-CPDs, in which at each bootstrap iteration we learn a tree-CPD on resampled data and in the end examine the number of times in which a rule was learned. Thus, after *N* bootstrap iterations we gather *N* tree-CPDs, and the confidence of each rule can then be estimated by the fraction of tree-CPDs that contain it (we used *N* = 1000). We repeated the bootstrap procedure without restricting the maximal depth of the learned tree, and with different constraints on the maximal depth of the leaves, from 1 to 6. Figure 4A–E shows the most confident rules that we learned with a confidence greater than 0.3. When the maximal depth was allowed to be greater than 5, no high confidence rules were learned. Figure 4F shows how all of these rules can be concisely combined into one single tree-CPD.

The fact that our approach extracted a set of rules with high confidence and that they can be concisely represented by a single decision tree demonstrates that we can indeed identify a subset of genes and interaction rules among them that predict neuronal connectivity. We next examined the specific set of gene clusters that were extracted in high confidence rules. Note that each cluster is represented by a single gene but may contain several genes. We examine all the genes in each cluster since our model cannot distinguish between them (see Discussion section).

The most confident cluster of genes that affect the chemical synapses is in the root of our resulting tree (Figure 4F). It is represented by the *hmr-1* gene. This cluster contains two genes that have a similar expression pattern in the neurons of the worm. These genes are *unc-55* and *hmr-1*. *Unc-55* encodes a nuclear hormone receptor. It was shown in [18] that *unc-55* is essential for the producing the synaptic pattern that distinguishes ventral D motor neurons from the dorsal D motor neurons. *Hmr-1* gene encodes two isoforms of a classical cadherin that contain extracellular cadherin and a highly conserved intracellular domain. Cadherin superfamily molecules are known to be involved in many biological processes, such as cell recognition, cell signaling, cell communication, morphogenesis, angiogenesis, and possibly even neurotransmission [19]. Furthermore, in humans, the Protocadherins, which are a subfamily of the Cadherin superfamily, have been proposed to constitute the molecular identifiers of Sperry's chemoaffinity hypothesis [9]. Indeed, this gene is predicted to function as a calcium-dependent, homophilic cell–cell adhesion receptor. It was also predicted to be required for mediating cell migrations and for fasciculation and outgrowth of a subset of motor neuron processes [20].

The *akt-1* gene cluster appears in the first level of the resulting tree in the context where the *hmr-1* gene is not expressed. It contains both the *akt-1* and the *akt-2* genes which encode an ortholog of the serine/threonine kinase Akt/PKB that functions to regulate processes such as dauer larval development and salt chemotaxis learning [21],[22]. In addition, they genetically interact with the insulin signaling pathway which was shown to be essential for ensuring that the nervous system is wired correctly during development in *Drosophila* [23]. The rest of the clusters that are part of high confident rules contain only one gene which is also the representative of these clusters.

In the context where the *hmr-1* gene is expressed we find the *npr-1* gene. It encodes a predicted G protein-coupled neuropeptide receptor that is homologous to the mammalian neuropeptide Y receptor. *Npr-1* affects some aspect of *unc-6*/netrin-mediated branching of motor neurons, as strong *npr-1* mutations can suppress abnormal migration of ventral nerve cord neurons induced by overexpression of *unc-6* lacking domain C [24].

As we continue to traverse over the resulting tree, we encounter the *cdh-3* gene next. It encodes a member of the cadherin superfamily. Unlike the *hmr-1* gene it encodes a nonclassical cadherin (fatlike cadherin) that has a very large extracellular region. *Cdh-3* was shown to affect morphogenesis of tail epithelia and excretory function [25]. *Cdh-4*, the only other fatlike cadherin gene in the *C. elegans* genome was shown to control axon guidance, cell migration and pharynx development [26].

Further down the tree, the *glr-1* gene encodes an AMPA ionotropic glutamate receptor subunit. *Glr-1* activity is required for mediating some behavioral responses [27]. Its expression is dependent on the homeodomain protein encoded by *unc-42* [28] that is required for axonal pathfinding of neurons. In wild-type worms, the axons of AVA, AVD, and AVE lie in the ventral cord, whereas in *unc-42* mutants, the axons are anteriorly, laterally, or dorsally displaced, and the mutant worms have sensory and locomotory defects [29].

The *osm-6* gene encodes a protein that is localized to cytoplasm, including processes and dendritic endings where sensory cilia are situated. Mutation in this gene causes defects in the ultrastructure of sensory cilia and defects in chemosensory and mechanosensory behaviors [30]. It was shown that sensory activity affects sensory axon development [31] and that disruptions to this activity may alter neuronal connectivity [32].

Finally, in the last level of our resulting tree we find the *unc-4* gene. It encodes a homeodomain transcription factor with orthologs in *Drosophila* and vertebrates. A mutation in the *unc-4* gene alters the pattern of synaptic input to one class of motor neurons in the *C. elegans* ventral nerve cord. It was shown that *unc-4* is required for establishing the identity of the A class motor neurons DA and VA, and is thus required for movement, axon guidance, and synapse formation [33].

Thus, examining the single tree that contains the rules that were extracted with high confidence (Figure 4F), we find that its set of genes or their orthologs in other species have all been previously implicated as having a direct or indirect role in neuronal connectivity, which combined with the robustness with which they are predicted in our tree, increases our confidence in their role in the process.

## Discussion

In this study we performed a systematic search for genes that mediate the last phase of chemical synaptic partner selection, while incorporating geometrical constraints on neuronal connectivity. We demonstrated that combination of expression patterns can be used to predict chemical synapse connectivity with good accuracy. We highlight specific genes and provide the combinatorial logic by which these genes may interact to specify the formation of a chemical synapse between neighboring neurons.

A key observation of our study is that neuronal wiring can be predicted by logical combination of a small number of genes. This finding was partly biased by the search for small decision trees but the fact that it achieves good accuracy supports its validity. An alternative design could have used hundreds or thousands of different genes to achieve the same connectivity, for example, one gene for each synapse. Our result is supported by the observation of White [2] that if a neuron is for some reason (mutation or variation between isogenic individual) created in a slightly different surrounding than usual with a slightly different set of close neurons, it creates a different set of synapses. If every synapse was encoded in the genome independently by an independent set of genes, this would not be the case. The modular design we find is similar to other biological systems, such as signal transduction cascades, where the mapping between signal inputs to the cells and their response in highly different pathways and cells is carried out by a small set of core modules [34]. It may be that this modular design, observed here in the context of neuronal wiring, is more optimal or evolvable than the alternatives. It also raises the possibility that the genetic mechanism for neuronal wiring in *C. elegans* is rather similar to the mechanism in more complex organisms, but this hypothesis should of course be reexamined when similar data becomes available for more complex organisms.

Despite its predictive power, our approach has several limitations. Currently, both the connectivity network and the gene expression pattern are crude and noisy [11] and some important pieces of information are missing. The most prominent limitation of our model is its inability to infer the causal relationship between gene expression and synapse formation. In the absence of temporal or interventional data, our model cannot distinguish between genes that are responsible for chemical synaptic specificity and genes that are over- or underexpressed in either side of a chemical synapse due to its formation. Another limitation of our model is that it cannot distinguish between genes that are directly responsible for synaptic specificity and genes that have only indirect affect on this process within the same gene cluster. This distinction can sometimes be made manually by examining the expression patterns of the genes in nonneuronal cells or by examining the relevant literature.

One of the strengths of our approach is that it can be easily extended to deal with many types of additional data. For example, the gene expression in individual cells is measured by GFP fluorescence or by immunostaining. These levels are of course not binary (on or off), but they appear as such in the single database that is currently available [11]. Future large-scale work could solve this problem by systematic detection of the continuous expression pattern of genes in a uniform way [35],[36]. By minor modifications to the tree-CPD representation and learning procedure, we can apply our method to learn nonbinary tree-CPDs and automatically detect the thresholds on the expression level by which a split should be made.

An interesting observation by White et al. [3] is that the neuron groups AVD, AVE and AVB all have extensive synapses onto AVA along the cord (each neuron group consists of neurons with similar morphologies and connectivity patterns and denoted by an arbitrary three-letter name [3]). However, in the nerve ring, processes from these cells do not form such synapses even though they are accessible to AVA (i.e. are adjacent to its processes). One possible explanation for this is time. It is possible that the genetic signal for synapse formation is changed at a specific time point during development and that this change affects only newer processes. Another possible explanation could be signals that are localized to specific regions of the cell. Knowing the specific time each synapse was created and the specific adjacent set of neurons in conjunction with the specific (preferably, intracellular) expression pattern of all the genes in the neighborhood at that specific time would lead to the most comprehensive and complete picture. All of this data could be easily incorporated into the data instances from which we learn with relatively minor changes. Such timing information may also address the problem of cause and effect that currently cannot be disentangled by our approach. Solving this problem would lead to the most convincing proof for the determination of neuronal wiring by gene expression patterns in *C. elegans*.

## Materials and Methods

### Data and preprocessing

This work combines two types of input data: the gene expression signature of the neurons and the synaptic connectivity network. For the Boolean single-cell gene expression signature of the neurons we have used the data provided by Varadan et al. [12]. This data was extracted from WormBase (http://www.wormbase.org version WS180), the main public repository of the *C. elegans*'s genetic data, using a stringent mining criteria and was manually curated.

The single-cell gene expression data in WormBase was gathered from many studies that read the GFP levels from transgenic worms in which a GFP gene was inserted downstream to the promoter of the investigated gene or stained the worm with a specific protein antibody in different developmental stages. This data is considered crude and noisy due to inaccuracies in the gathering process of the data from the animal and due to its discretization into a Boolean expression of “on” and off”.

As a preprocess stage we eliminated all the genes that were expressed in less than 2% of the neurons since they carry little information for our computation. In order to avoid instability of the results due to genes that have very similar expression pattern over the neurons, the remaining 251 genes were clustered using hierarchical clustering. First the Hamming distance (the percentage of neurons that disagree on the expression) between every pair of expression patterns was calculated, then a nearest neighbors algorithm was used to construct a linkage tree. This tree was divided into 133 expression classes by applying a cutoff of 0.8 to the inconsistency coefficient [37] of its edges. The average Hamming distance between different genes in the same class was 1.7% and only 5% of the expression classes contain more than 4 genes. The typical expression pattern of an expression class that contains more than one gene was set to be the same as the expression pattern of the gene that has the minimum average Hamming distance from all other genes in this class. The final gene set and their assignment to expression classes are listed in Table S1.

For the synaptic connectivity network we used a version of the pivotal works of White et al. [3] and Hall and Russell [38] that was recently compiled by Chen et al. [39]. This version contains the complete connectivity of 280 nonpharyngeal neurons and it is publicly available at Wormatlas (http://www.wormatlas.org/). We have used this synaptic connectivity network to build the set of weighted data instances from which we learn our model. The weight of a positive data instance (i.e. data instance for positive example) is proportional to the number of chemical synapses that were observed in this direction, whereas the weight of the negative data instance is set to 1. The biological motivation for the use of weights is that the number of identical synapses in the same direction is positively correlated with its invariability between isogenic individuals. Specifically, some of the small, single synapses are not present in some individuals and therefore may be less significant [3] while on the other hand a broad core of connections that are constant in all the individuals in the population includes most of the strong synaptic connections containing many synapses [1].

To obtain balance between the weights of the positive and the negative data instances, the weights of the positive data instances were normalized such that their sum would equal the sum of weights of the negative data instances. As a result, the final data instances set contained 4574 weighted examples composed of 48% positive and 52% negative, each carrying 50% of the total weights.

### Learning the model

Learning the tree-CPD model from the input data requires two components. The first is a scoring scheme that measures the goodness of fit of the model and enables the comparison of two different models. The scoring method that we used is the Bayesian score [15]. This score is a standard and a principled way to tradeoff model complexity and fit to data, thus it relaxes the necessity of Varadan et al. in [12] to predetermine the number of expected interacting genes. For detailed explanation about the Bayesian score and comparison to the maximum likelihood score which is a scoring method that does not tradeoff model complexity and fit to the data see Text S2 and Figure S3.

The second component that is required is a search heuristic to scan the exponentially large model space in order to find the highest scoring model. We have adopted the approach of Friedman and Goldszmidt [14] which was inspired by Quinlan and Rivest [40]. According to this approach, the tree is learned in two phases. In the first phase, the tree is grown in a top-down fashion, starting from the trivial empty tree and growing till the maximal tree is learned. In each step of this phase, we split one leaf of the tree using the variable that induces the best scoring tree. During this process there might be some splits that will reduce the score of the tree, but we do not stop if it happens, since further growth of the tree might compensate for this temporary reduction of the score. In the second phase, we trim the tree in a bottom-up manner. We start from the leaves and climb to the root, checking for each inner node of the tree if the replacement of the subtree rooted at it with an empty tree will increase the score. If it does, we trim the tree at that node and continue. The downhill splits we are willing to take during the first phase prevent the learning process from getting stuck at every local minima of the search space, like most of the greedy search heuristics for learning decision trees [14].

We have used the standard boosting algorithm AdaBoost introduced by Freund and Schapire in 1995 [16] to improve the classification accuracy of the tree-CPD. The main idea of AdaBoost is to change the weights of the training data according to the success in their classification. In each round, the weights of incorrectly classified examples are increased so that in the next round, the tree-CPD has to focus on the hard examples. The final combined classifier is a weighted majority vote of all the tree-CPDs from all the iterations. A pseudocode that summarizes this procedure is given in Protocol S1. An important advantage of AdaBoost compared to other methods such as neural networks and support vector machines is that it works well without fine tuning and no sophisticated nonlinear optimization is necessary. It also tends not to overfit the data [41],[42]. In fact, Adaboost in conjugation with decision trees was described as the best “off-the-shelf” classifier in the world [41].

### Evaluating the model

The performance of the model was measured using a standard 5-fold cross-validation scheme. In this procedure, we randomly partitioned the data into five equal parts. We then made some small adjustments to the partition in order to eliminate dependencies as described below and learned a model on each of the five subsets of four parts and tested its performance on the held out subset. The final performance estimator is an average of the performance of the five estimators obtained.

To avoid dependencies between the train and test sets that might bias the results, the partition of the data into train and test sets must consider the symmetries of the connectivity diagram of *C. elegans* since symmetrical neurons tend to form similar connections [1] and often express similar sets of genes. The main symmetry axis in the worm is the left–right axis and the secondary symmetry axis which appears especially in the pharynx is the dorsal–ventral axis. Thus, for some neurons there is even a 6-fold symmetry! In addition, for several neurons (especially for motorneurons) there is longitudinal duplication throughout the ventral and dorsal cord. The nomenclature of the neurons suggested by white et al. [3] captures these symmetries. E.g. the IL1 group of neurons consists of the symmetrical neurons: IL1DL, IL1DR, IL1L, IL1R, IL1VL and IL1VR. The last two letters show the symmetry where D, V, L, and R stand for Dorsal, Ventral, Left, and Right, respectively.

To eliminate the dependence of the train and test sets, Kaufman et al. [11] used only the neurons from right side of the worm. However, this approach does not eliminate dependencies of the dorsal–ventral symmetry axis and the amount of data that remains for learning is reduced significantly. We have used a different approach, in which if (X,Y) is an example in the train set than every pair of (X′,Y′), (X′,Y) and (X,Y′) will also be in the train set, where X′ and Y′ are neurons that were assigned by white et al. to the same group of neurons as X and Y, respectively. This approach uses all the data and eliminates the bias that might be caused by the known symmetries.

Prediction accuracy of the model was measured by the standard area under the receiver operating characteristic (ROC) curve. The ROC curve plots the fraction of true positives versus the fraction of true negatives for a binary classifier, while its discrimination threshold varies. The area under the ROC curve (AUC) is a measure that intuitively can be interpreted as the probability that when we randomly pick one positive and one negative example, the classifier will assign a higher score to the positive example than to the negative one.

Statistical significance of the prediction performance was calculated against two empirical null distributions: the shuffled expression and the shuffled connectivity distributions. The first was constructed by repeating the prediction procedure 50 times, each time with neuronal identities reshuffled. This empirical null distribution was used in previous studies [11],[12]. The motivation behind this test is to evaluate whether the prediction accuracy obtained for the real data can be attributed to real dependence between the expression profiles of the neurons and synaptic connectivity, or if it is a result of the properties of the input data such as the number of different expression patterns, the degree distribution of the network, etc. Indeed, the best AUC that was achieved for this empirical null distribution was 0.63 (Figure 3). This AUC is significantly above the 0.5 score that a pure random guess would achieve. This means that even if there was no real relation between gene expression and chemical synapse formation, it is possible to find a model that is this good just by chance due to the properties of the input data. To better understand this, think of the extreme case of a starlike network in which there is one neuron that is postsynaptic to all other neurons in the network and that there are no other synapses in the network. If, after the shuffling of the identities of the neurons, this single neuron expresses a gene X that no other neuron expresses (it is not unreasonable if there are enough, different, gene expression patterns) then the rule: “if a neuron expresses gene X than it will be postsynaptic to every other neuron in its neighborhood” will have strong evidence in both the train and the test sets, regardless of the partition of the examples into train and test. As a consequence the classifier that is learned on the train set will achieve AUC that is greater than 0.5 on the test set, even though the identities of the neurons were shuffled.

The second distribution was constructed by repeating the prediction procedure 50 times, each time with the signs of the examples reshuffled, while maintaining the same amount of positive and negative examples for each neuron. In other words, each neuron chooses to create a chemical synapse to a random subset of the neurons in its neighborhood while the size of this random set is equal to the number of neurons it chooses in the real data. The motivation behind this distribution is to test whether or not each neuron chooses to form synapses with a subset of its neighboring neurons based on their gene expression profile. The significance of the result with respect to this second empirical null distribution is generally lower (Figure 3), since much of the relation between gene expression and synaptic connectivity from the real data is maintained due to the limited shuffling (there is a correlation of ∼0.6 between the real data and each shuffled data from this distribution).

To evaluate the confidence of the rules that we learned we used a nonparametric Bootstrap. According to this method, we generated many resampled versions of the data and learned a model from them. This way we collected many reasonable models for the real data. The confidence of a rule is the percentage of models that agree with it. Each resampled version of the data was generated by resampling the data instances with replacement for *m* times, where *m* is the number of data instances in the data, therefore it is expected to contain about 63.2% of the data instances and the rest are duplicates. A pseudocode that summarizes this procedure is given in Protocol S2.

The confidence of complex rules tends to be smaller relative to simpler rules due to several reasons: First, the deeper the tree-CPD, the larger the search space is and the probability to learn exactly the same rules in different bootstrap iterations decreases. Second, decision trees are inherently unstable [41], i.e. slight perturbation of the data may lead to a different learned tree especially when the tree is deep. Third, the gene expression data is highly correlated. Although we aggregated highly correlated gene expressions into expression classes there still exists correlation between these expression classes. Closely related expression classes may switch roles in tree-CPDs that are learned on different resampling of the data.

## Supporting Information

Text S1 Comparison between the Performance of Boosted and Nonboosted Tree-CPDs.
(0.03 MB DOC)

Text S2 The Bayesian Score and the Maximum Likelihood Score.
(0.06 MB DOC)

Protocol S1 Pseudocode for Boosting Tree-CPD Using AdaBoost.
(0.07 MB DOC)

Protocol S2 Pseudocode for Confidence Evaluation Using Nonparametric Bootstrap.
(0.03 MB DOC)

Figure S1 Summary of the Prediction Performance as a Function of the Maximal Depth of the Tree-CPD without Boosting. The depth of a tree-CPD with unconstrained maximal depth is determined automatically by the Bayesian score and the tree-CPD constructing heuristic. Standard deviation of the real data was calculated on 50 iterations of 5-fold cross validation, each time for a different division of the data to train and test sets. Standard deviation of the random models was calculated on 50 iterations of 5-fold cross validation, each time for a different shuffling of the data.
(0.17 MB TIF)

Figure S2 The Prediction Performance of a Boosted Tree-CPD with Maximal Depth of 2 as a Function of the Number of AdaBoost Iterations. Standard deviation of the real data was calculated on 50 iterations of 5-fold cross validation, each time for a different division of the data to train and test sets. Standard deviation of the random models was calculated on 50 iterations of 5-fold cross validation, each time for a different shuffling of the data. Similar results are obtained for different maximal depths of tree-CPD (data not shown).
(0.21 MB TIF)

Figure S3 Classifier Learned with Bayesian Score Is Less Prone to Overfitting Than Classifier Learned with Maximum Likelihood Score. Comparison between the performance on the train and test sets of tree-CPD classifier that was learned using the maximum likelihood score (left) and to that was learned using the Bayesian score (right) as a function of the maximal depth of the leaves. The performance is measured as the percentage of correctly classified examples. Standard deviation was calculated on 50 iterations of 5-fold cross validation, each time for a different division of the data to train and test sets.
(0.26 MB TIF)

Table S1 Assignment of Genes to Expression Classes.
(0.03 MB XLS)
