# Supplementary material for: Using Expression Profiles of Caenorhabditis elegans Neurons To Identify Genes That Mediate Synaptic Connectivity
Source: PLoS Comput Biol. 2008 Jul 11;4(7):e1000120. doi: 10.1371/journal.pcbi.1000120 (PMC2517614; doi:10.1371/journal.pcbi.1000120)
Supplement: Text S1 — Comparison between the Performance of Boosted and Nonboosted Tree-CPDs. (0.03 MB DOC) [file pcbi.1000120.s001.doc]

**Text S1: Comparison between the performance of boosted and non-boosted tree-CPDs**

Figure S1 shows the prediction performance of the best, single, tree-CPD that was learned as a function of the external constraint on the maximal depth of the leaves. The real data achieved significantly better results than the two empirical null distributions when the tree was allowed to grow beyond the depth of two. This significance is increased as the maximal depth is increased. The biological meaning of this is that the chemical synapse formation is affected by the joined expression of several genes (probably more than two) in a complex way.

Comparing the results from Figure 3 and Figure S1 reveals that any boosted tree-CPD performed better than the best tree-CPD that was not boosted. This behavior demonstrates the power of boosting, which was able to boost the performance of even the weakest classifier to a final combined classifier that is stronger than the strongest single classifier. It also implies that even our best non-boosted model does not reveal all the complex relationships between genes and chemical synapse formation.

The performance of boosted tree-CPDs seems independent of the maximal depth of their leaves, in contrast to the non-boosted case. We can speculate that the reason for this is that the boosted case achieved the maximal possible prediction performance given the input data, which is around 0.82 regardless of the strength of the weak classifier.
