# Supplementary material for: Using Expression Profiles of Caenorhabditis elegans Neurons To Identify Genes That Mediate Synaptic Connectivity
Source: PLoS Comput Biol. 2008 Jul 11;4(7):e1000120. doi: 10.1371/journal.pcbi.1000120 (PMC2517614; doi:10.1371/journal.pcbi.1000120)
Supplement: Text S2 — The Bayesian Score and the Maximum Likelihood Score. (0.06 MB DOC) [file pcbi.1000120.s002.doc]

**Text S2: The Bayesian score and the maximum likelihood score**

Here we present two scoring methods – the maximum likelihood score and the Bayesian score and explain why we chose the later. The maximum likelihood score is based on the probability of the data given a tree-CPD with parameters equal to their maximum likelihood estimators, formally:

$$Score_{L}(T:I)=\log P(I|T,\theta_{T}^{'})$$

where *I* is the set of data instances, *T* is a tree-CPD and *θ’_T_* are the set of maximum likelihood estimators for the parameters of *T*. This score is also proportional to the mutual information of the data and the tree-CPD minus the entropy of the data, under the distribution imposed by the maximum likelihood estimators [1], formally:

$$\hat{P}$$

Where is the distribution of chemical synapse formation imposed by the decomposition of *I* over *T*. For a complete tree, this score is equivalent to the conditional entropy measure that was used by Varadan et al. in [2] to search for a group of genes that minimize the uncertainty of the existence of synapses. However, this score tends to overfit the data, since almost every addition of nodes to the tree will increase this score as the uncertainty of the existence of synapses is decreased. Thus it is necessary to pre-determine the number of expected interacting genes, as was done by Varadan et al. in [2].

The second scoring method is the Bayesian score [3]. This score is proportional to the posterior probability of the tree given the data, formally,

$Score_{B}(T:I)=\log P(T)+\log P(I|T)$

where the first term of the score depends on the probability of the structure of the tree-CPD and the second term is equal to

$$P(I|T)=\int_{\theta_{T}} P(I|T,\theta_{T})P(\theta_{T}|T)d\theta_{T}$$

This second term evaluates the fit of the model to the data by averaging the likelihood of the data over all possible parameterization of the model. In addition it enables the incorporation of prior knowledge over the parameters of the model. The most simple and intuitive priors are the Dirichlet priors. These priors are specified by a set of hyperparameters corresponding to the number of imaginary examples we saw before starting the experiment. When the data is fully observed (like in our case) and the priors are in Dirichlet form this score has a simple analytic form as a function of the sufficient statistics of the model and the hyperparameters of the Dirichlet priors [3]. In our case, since we do not have prior knowledge for the structure of the tree nor for its parameters, we have used a uniform prior for the space of trees, and Dirichlet priors with all the hyperparameters equal to one for the parameters of the tree.

$W\to\infty$At the limit of the Bayesian score is equal to the Bayesian information criteria (BIC) [4,5]:

Where *W* is the sum of weights of *I* and *Dim(T)* is proportional to the number of parameters of *T*. The meaning of this is that Bayesian score is proportional to the Likelihood score minus a factor that is proportional to the dimensionality of the model, thus it exhibits a tradeoff between fit to data and complexity. As W grows, more emphasis is given to the fit to the data. This way, the use of the Bayesian score relaxes the demand for pre-determining the number of expected interacting genes.

Figure S3 compares the prediction performance of the tree-CPDs that were learned using a Bayesian scoring method to those of tree-CPDs that were learned using the maximum likelihood scoring method. In both of the scoring methods the performance on the train set of deep tree-CPDs was better than the performance of tree-CPDs that were restricted to a shallower maximal depth of the leaves. However, using the maximum likelihood score, tree-CPDs that were learned without restriction on the maximal depth of the leaves performed worse than tree-CPDs that were learned with this restriction. This was not the case when the Bayesian score was used, thus demonstrating how classifier learned with Bayesian score is less prone to overfitting than classifier learned with maximum likelihood score.

**References**

1. Cover TM, Thomas JA (2006) Elements of information theory. Hoboken, N.J.: Wiley-Interscience. xxiii, 748 p. p.

2. Varadan V, Miller DM, 3rd, Anastassiou D (2006) Computational inference of the molecular logic for synaptic connectivity in C. elegans. Bioinformatics 22: e497-506.

3. Heckerman D, Geiger D, Chickernig D (1995) Learning Bayesian Networks: The Combination of Knowledge and Statistical Data. Machine Learning 20: 197-243.

4. Haughton DM-A (1988) On the choice of a model to fit data from an exponential family. The Annals of Statistics 16: 342-355.

5. Schwarz G (1978) Estimating the dimension of a model. The Annals of Statistics 6: 461-464.
