# Supplementary material for: Using Expression Profiles of Caenorhabditis elegans Neurons To Identify Genes That Mediate Synaptic Connectivity
Source: PLoS Comput Biol. 2008 Jul 11;4(7):e1000120. doi: 10.1371/journal.pcbi.1000120 (PMC2517614; doi:10.1371/journal.pcbi.1000120)
Supplement: Protocol S1 — Pseudocode for Boosting Tree-CPD Using AdaBoost. (0.07 MB DOC) [file pcbi.1000120.s003.doc]

**Protocol S1. Pseudo-code for boosting tree-CPD using AdaBoost**

$X_{i}post$ $S_{i}$ $W_{i}$ $X_{i}pre$Given: a set *I* of *m* data instances, where each data instance is composed of a pre-synaptic neuron , a post-synaptic neuron , a weight , and a flag that is equal to 1 if there exists a chemical synapse in this direction or -1 otherwise.

$W_{i}$set D_1_(i) =

For *n* = 1,….,*N*:

- Learn a tree-CPD for distribution *D_n_* using the Bayesian score and a two phase construction heuristic:

$$h_{n}:(Xpre,Xpost)\to\left\{-1,+1\right\}$$

- The learned tree-CPD is the weak hypothesis with error:

$$\varepsilon_{n}=\Pr_{i\sim D_{n}}\left[h_{n}\left(X_{i}pre,X_{i}post\right)\neq S_{i}\right]$$

$$\alpha_{n}=\frac{1}{2}\ln(\frac{1-\varepsilon_{n}}{\varepsilon_{n}})$$

- Choose
- Update

$$=\frac{D_{n}(i)\exp(-\alpha_{n}S_{i}h_{n}(X_{i}pre,X_{i}post))}{Z_{n}}$$

where *Z_n_* is a normalization factor (chosen so that *D_n+1_* will be a distribution).

- Output the final hypothesis:

$$H(Xpre,Xpost)=sign(\sum_{n=1}^{N} \alpha_{n}h_{n}(Xpre,Xpost)$$
