# Supplementary material for: Using Expression Profiles of Caenorhabditis elegans Neurons To Identify Genes That Mediate Synaptic Connectivity
Source: PLoS Comput Biol. 2008 Jul 11;4(7):e1000120. doi: 10.1371/journal.pcbi.1000120 (PMC2517614; doi:10.1371/journal.pcbi.1000120)
Supplement: Protocol S2 — Pseudocode for Confidence Evaluation Using Nonparametric Bootstrap. (0.03 MB DOC) [file pcbi.1000120.s004.doc]

**Protocol S2. Pseudo-code for confidence evaluation using nonparametric**

**Bootstrap**

Given: a set *I* of *m* data instances.

For i = 1,….,*N*:

- Generate a resampled data set by re-sampling with replacement *m* times. Set the weight of every data instance to be its initial weight times the number of times that it was sampled. Denote the resulting weights distribution by D_i_.
- Learn a tree-CPD T_i_ for distribution D_i_ using the Bayesian score and the two phase construction heuristic.

For each rule r (leaf in a tree) calculate:

$$confidence(r)=\frac{1}{N}\sum_{i=1}^{N} r(T_{i})$$

Where *r*(*T*) is 1 if r is a rule in *T*, and 0 otherwise.

Note that since we compare leafs of different trees, *r* should be insensitive to the order of the genes in the rules. For instance, these two rules are identical:

1. If hmr-1 is expressed in the post synaptic neuron and npr-1 is not expressed in the pre synaptic neuron than the probability for a chemical synapse formation in this direction is high.

2. If npr-1 is not expressed in the pre synaptic neuron and hmr-1 is expressed in the post synaptic neuron than the probability for a chemical synapse formation in this direction is high.
