# Supplementary figures and images for: Using Expression Profiles of Caenorhabditis elegans Neurons To Identify Genes That Mediate Synaptic Connectivity
Source: PLoS Comput Biol. 2008 Jul 11;4(7):e1000120. doi: 10.1371/journal.pcbi.1000120 (PMC2517614; doi:10.1371/journal.pcbi.1000120)

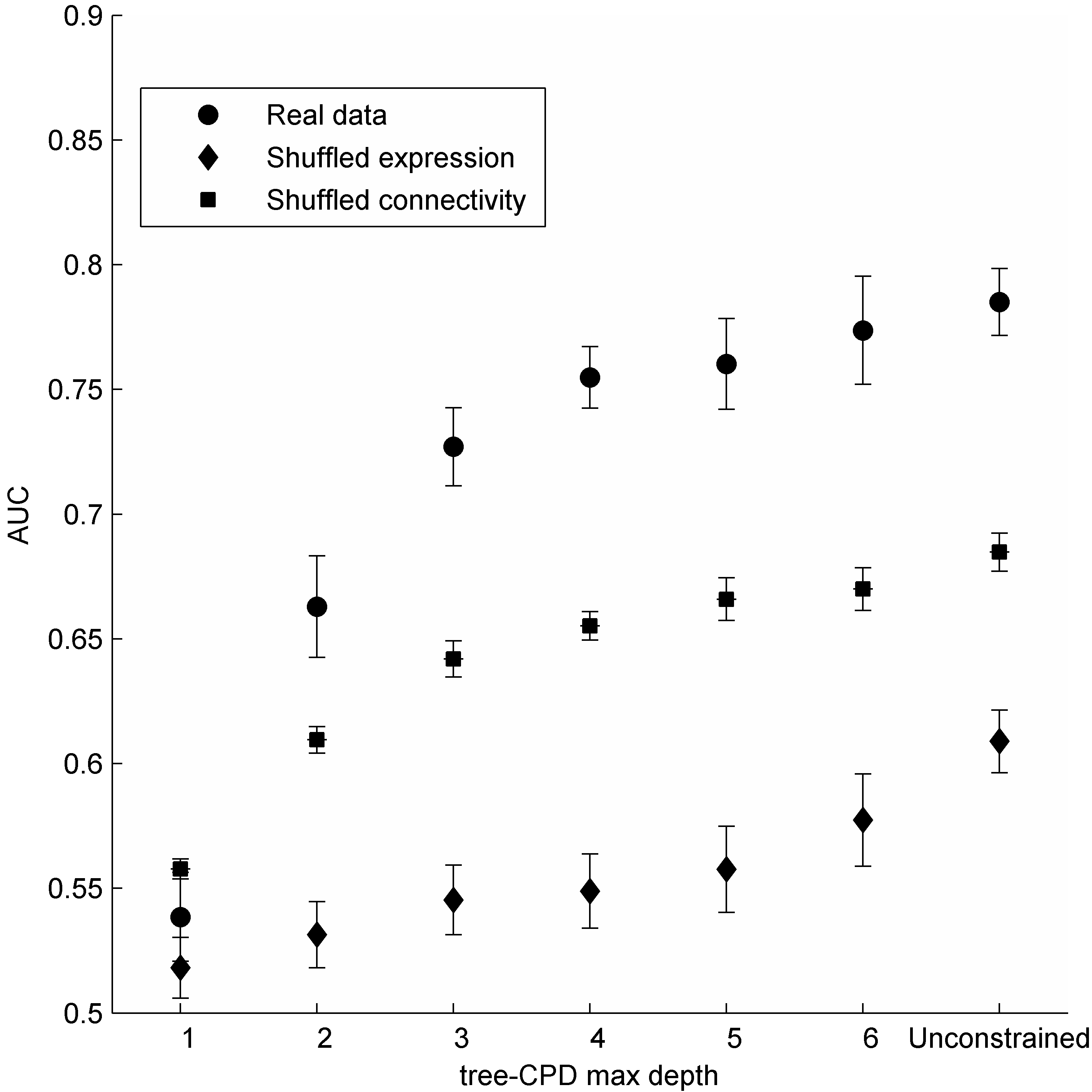

Supplement: Figure S1 — Summary of the Prediction Performance as a Function of the Maximal Depth of the Tree-CPD without Boosting. The depth of a tree-CPD with unconstrained maximal depth is determined automatically by the Bayesian score and the tree-CPD constructing heuristic. Standard deviation of the real data was calculated on 50 iterations of 5-fold cross validation, each time for a different division of the data to train and test sets. Standard deviation of the random models was calculated on 50 iterations of 5-fold cross validation, each time for a different shuffling of the data. (0.17 MB TIF) [file pcbi.1000120.s005.tif]

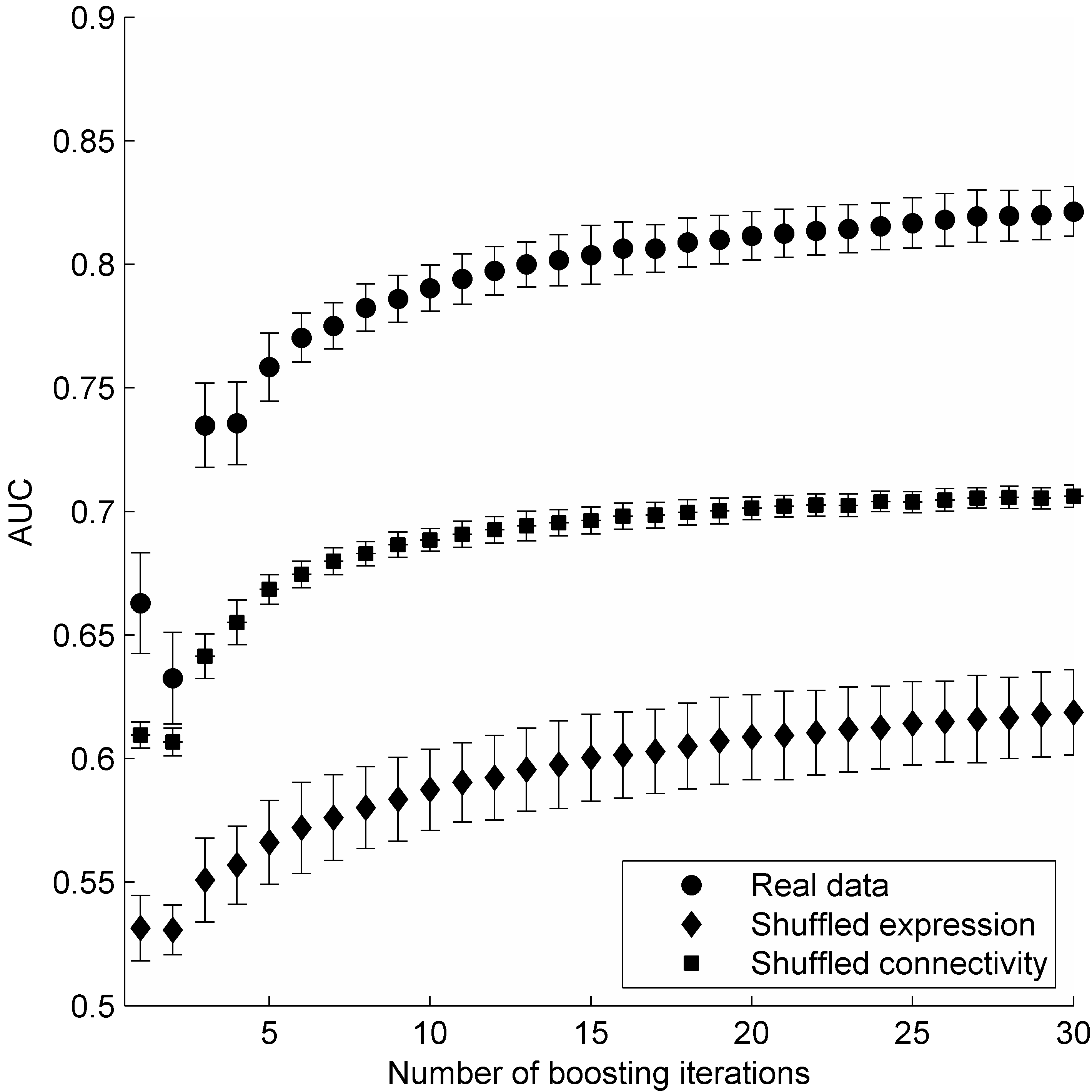

Supplement: Figure S2 — The Prediction Performance of a Boosted Tree-CPD with Maximal Depth of 2 as a Function of the Number of AdaBoost Iterations. Standard deviation of the real data was calculated on 50 iterations of 5-fold cross validation, each time for a different division of the data to train and test sets. Standard deviation of the random models was calculated on 50 iterations of 5-fold cross validation, each time for a different shuffling of the data. Similar results are obtained for different maximal depths of tree-CPD (data not shown). (0.21 MB TIF) [file pcbi.1000120.s006.tif]

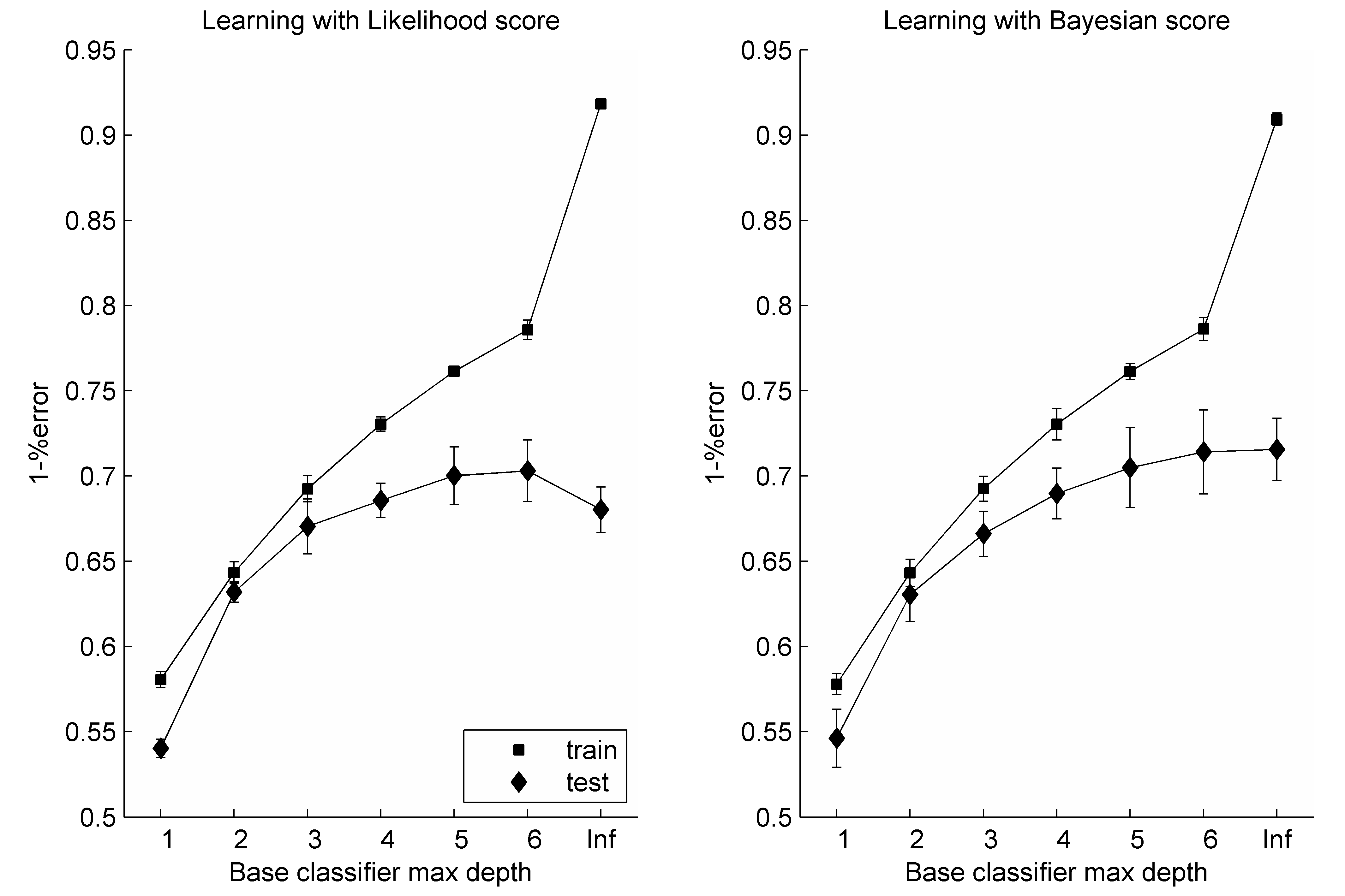

Supplement: Figure S3 — Classifier Learned with Bayesian Score Is Less Prone to Overfitting Than Classifier Learned with Maximum Likelihood Score. Comparison between the performance on the train and test sets of tree-CPD classifier that was learned using the maximum likelihood score (left) and to that was learned using the Bayesian score (right) as a function of the maximal depth of the leaves. The performance is measured as the percentage of correctly classified examples. Standard deviation was calculated on 50 iterations of 5-fold cross validation, each time for a different division of the data to train and test sets. (0.26 MB TIF) [file pcbi.1000120.s007.tif]
